# Scurvy may occur even in children with no underlying risk factors: a case report

**DOI:** 10.1186/s13256-020-2341-z

**Published:** 2020-01-24

**Authors:** Romina Gallizzi, Mariella Valenzise, Stefano Passanisi, Giovanni Battista Pajno, Filippo De Luca, Giuseppina Zirilli

**Affiliations:** Department of Human Pathology in Adult and Developmental Age “Gaetano Barresi”, University of Messina, Via Consolare Valeria 1, 98124 Messina, Italy

**Keywords:** Anemia, Ascorbic acid, Gingival bleeding, Hematochezia, Musculoskeletal pain, Petechial hemorrhages

## Abstract

**Background:**

Since ancient times, scurvy has been considered one of the most fearsome nutritional deficiency diseases. In modern developed countries, this condition has become very rare and is only occasionally encountered, especially in the pediatric population. Underlying medical conditions, such as neuropsychiatric disorders, anorexia nervosa, celiac disease, Crohn disease, hemodialysis, and severe allergies to food products may enhance the risk of developing scurvy.

**Case presentation:**

We report the case of an otherwise healthy 3-year-old white boy who developed scurvy due to a selective restrictive diet derived from his refusal to try new food. He presented to our clinic with asthenia and refusal to walk. During hospitalization he developed severe anemia and hematochezia. A diagnosis of scurvy was assessed on the basis of nutritional history, clinical features, radiographic findings, and laboratory findings. Supplementation of ascorbic acid enabled a prompt resolution of symptoms.

**Conclusions:**

Scurvy is caused by vitamin C deficiency. Cutaneous bleeding, mucosal bleeding, and anemia represent typical manifestations of the disease. These symptoms are directly connected to ascorbic acid involvement in collagen biosynthesis. Some radiographic findings can be useful for the diagnosis. Treatment aims to normalize serum levels of vitamin C in order to counteract the deprivation symptoms. The present case report demonstrates that scurvy may sporadically occur in pediatric patients, even in individuals with no predisposing medical conditions and/or potential risk factors.

## Background

Scurvy, also known as vitamin C deficiency, is an ancient disease that has existed for more than 3 millennia [1]. In modern developed countries, this condition has become very rare and may only occasionally be encountered; it is mainly associated with underlying comorbidities and risk factors [2–4].

Sporadic cases of scurvy are still observed, primarily among older and indigent persons who live alone and prepare their own food, as well as in alcoholics and food faddists [5].

In the pediatric population, scurvy is even more uncommon, at least in individuals with no underlying medical conditions [5]. Until recently, in developed countries, the occurrence of scurvy in children had become a historical footnote, with most radiologists having never encountered a case [6].

In the last few years, case reports describing the occurrence of scurvy in children with autism or other neuropsychiatric disorders have become less infrequent [7–17]. Additional at risk groups include children with iron overload (such as from multiple blood transfusions in sickle cell anemia or thalassemia), anorexia nervosa, celiac disease, Crohn disease, hemodialysis, and severe allergies to food products [18] or other causes of restricted dietary intake, such as fructose intolerance [6].

In the present study we describe the history of an otherwise healthy child with scurvy and none of the above reported risk factors, in order to highlight a rare disease which still exists in the pediatric population and may present even in individuals without neurological abnormalities or other underlying medical conditions. The aim of this case description was to underline the importance of recognizing vitamin C deficiency as a cause of hematochezia and severe anemia in a child without bleeding diathesis.

## Case presentation

A 3-year-old white boy was admitted to our clinic due to the following symptoms that had presented some weeks earlier: asthenia, diffuse lower-extremity musculoskeletal pain, and refusal to walk. His past medical history was unremarkable for developmental delay, neurologic disorders, and/or other underlying diseases. He had received the recommended and compulsory vaccinations according to Italian regulations. There was no recent history of fever, weight loss, trauma, bruising, and/or arthropathies.

At the time of admission to our clinic, a physical examination evidenced normal growth parameters and blood pressure. He presented a stature of 92.1 cm (17th percentile of expected height for age and sex) and a weight of 11.9 kg (3rd percentile of expected weight for age and sex). No dysmorphic features and no neurological or cardiovascular abnormalities were noted. After 2 days, he developed a clinical picture characterized by severe pallor, petechial hemorrhages on his arms and legs, gum hypertrophy and bleeding, macroscopic hematochezia and, finally, tachycardia and dyspnea, which required oxygen therapy. He exhibited markedly swollen, purple, and spongy gingivae, which bled spontaneously. There were no hepatosplenomegaly or lymphadenopathies or joint effusions.

At that time laboratory investigations revealed hemoglobin of 5.4 g/L, reticulocyte count of 3.5%, white blood cell count of 8.75 × 10^9^/L with a normal differential count, and a platelet count of 369 × 10^9^/L. His C-reactive protein serum levels, coagulation profile, muscle enzyme and electrolyte levels, and liver and kidney function tests were all within reference ranges for our laboratory. Thus, the child underwent a blood transfusion due to his severe anemia.

**Fig. 1:**
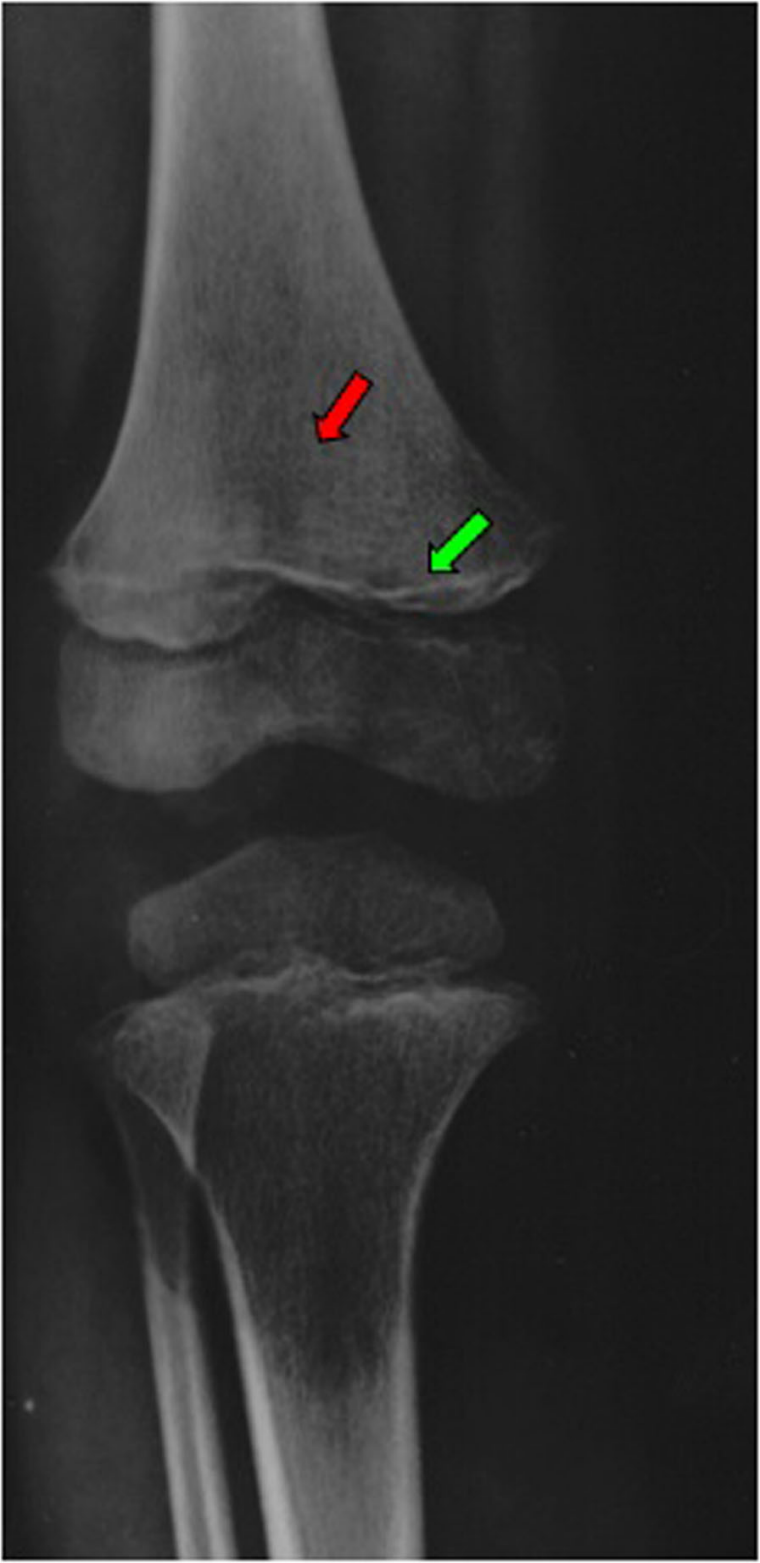
Right leg X-ray showing dense line at the distal femoral metaphysis (*green arrow*) and a lucent metaphyseal band (*red arrow*)

A skeletal survey revealed a dense line at the distal right femoral metaphysis and a lucent metaphyseal band, two findings which were felt to be suggestive of scurvy (Fig. 1). Such a diagnostic suspicion was, overall, supported by the constellation of gingival disease, purpura, anemia, hematochezia, musculoskeletal pain, and bone radiologic findings, as well as by the specific nutritional history. This revealed a strictly selective diet since the time of weaning, exclusively based on water, milk, and pasta, with complete avoidance of meat, fruit, vegetables, and fish for at least 2 years. Such inadequate eating habits derived from the boy’s refusal to try new food. A psychological evaluation highlighted a food behavioral disorder as a possible cause of his selective diet. No vitamin supplementation was administered. A review of the nutritional content of his diet revealed no dietary source of vitamin C and limited amounts of iron and vitamin D (Table 1). Nutritional laboratory investigations showed low serum concentrations of ascorbic acid, ferritin, iron, 25-hydroxyvitamin D (25-OH-vitamin D), and 1,25-hydroxyvitamin D (1,25-OH-vitamin D) (Table 2). On the basis of his nutritional history, clinical examination, radiographic findings, and laboratory findings, a diagnosis of scurvy was definitively assessed and vitamin C supplementation was added, starting from 300 mg/day for 28 days, followed by 100 mg/day in a maintaining phase.

**Table 1:** Specific nutritive intake of our patient* and recommended intake for age^§^

	Vitamin C	Vitamin D	Iron
Milk (3 cups daily)	7.5 mg	unknown	1.5 mg
Pasta (120 g daily)	0	0	0.8 mg
Daily recommended nutrient intake for age	35 mg	15 μg	8 mg

* According to the data of Reference [19]

^§^ According to the recommendations of Reference [20]

**Table 2:** Results of patient’s laboratory tests

Date	Laboratory test	Patient’s value	Reference range*
22 Jul	Hemoglobin (g/L)	5.4	11–15
22 Jul	Red blood cell count (× 10^12^/L)	1.92	3.5–4.5
22 Jul	Reticulocyte count (%)	3.5	0.5-2.5
22 Jul	White blood cell count (× 10^9^/L)	8.75	6–12
22 Jul	Neutrophil count (%)	54	60–70
22 Jul	Lymphocyte count (%)	40	20–35
22 Jul	Platelet count (×10^9^/L)	369	150–350
22 Jul	Activated partial thromboplastin time (seconds)	30.2	21–35
22 Jul	Plasma fibrinogen (g/L)	2.79	2–4
22 Jul	Prothrombin (%)	71	70–120
22 Jul	Creatine phosphokinase (U/L)	43	0–200
22 Jul	Lactate dehydrogenase (U/L)	432	150–460
22 Jul	Alanine transaminase (U/L)	12	0–50
22 Jul	Aspartate transaminase (U/L)	31	0–42
22 Jul	Creatinine (mg/dl)	0.2	< 1.2
22 Jul	Blood urea nitrogen (mg/dl)	18.5	0–20
22 Jul	Iron (μmol/L)	2.1	9–27
22 Jul	Ferritin (μg/L)	9.6	22–400
22 Jul	C-reactive protein (mg/L)	4.2	0–5
24 Jul	Ascorbic acid (μmol/L)	< 5	11–85
24 Jul	25-OH-vitamin D (nmol/L)	17	25–90
24 Jul	1,25-OH-vitamin D (pmol/L)	35	40–140

*1,25-OH-vitamin D* 1,25-hydroxyvitamin D, *25-OH-vitamin D* 25-hydroxyvitamin D, * for our hospital laboratory

During the subsequent weeks we recorded a progressive improvement in his general condition, with resolution of cutaneous and mucosal bleeding, weight gain, pain reduction, and amelioration in walking impairment. After 90 days of treatment with vitamin C, ascorbic acid levels increased to 59 μmol/L, which confirmed the diagnosis of scurvy. The subsequent normalization of radiologic findings corroborated this diagnosis (Table 3). Psychological support was mandatory in order to facilitate our patient’s acceptance of the renewed nutritional regimen.

**Table 3:** Timeline of medical history and interventions

	Summaries from initial and follow-up visits	Diagnostic testing	Interventions
T = 0 (20 Jul–2 Aug)	Physical examination: severe pallor, petechial hemorrhages on arms and legs, gum hypertrophy and bleeding, macroscopic hematochezia, and, finally, tachycardia and dyspnea. No evidence of hepatosplenomegaly or lymphadenopathies or joint effusions	Leg X-ray (21 Jul): dense lines at the distal right femoral metaphysis and a lucent metaphyseal band Laboratory tests (22–24 Jul): severe anemia, low levels of iron, ferritin, ascorbic acid, 25-OH-vitamin D, and 1,25-OH-vitamin D	Vitamin C supplementation was practiced, starting from 300 mg/day for 28 days, followed by 100 mg/day in maintaining phase. Introduction of fruit and vegetables in the child’s diet
T = 3 months (3 Nov)	Physical examination: good clinical state. No cutaneous or mucosal alterations. Resolution of limping	Laboratory tests: normal value of hemoglobin (12.6 g/dl) and ascorbic acid (59 μmol/L) Leg X-ray: no radiological anomalies	Suspension of any treatment

*1,25-OH-vitamin D* 1,25-hydroxyvitamin D, *25-OH-vitamin D* 25-hydroxyvitamin D

## Discussion

An unusual aspect of the present case was the absence of any potential risk factors, which demonstrates that scurvy may still be occasionally encountered in the pediatric population, even in individuals with no predisposing disorders, living in families without unusual eating habits.

Vitamin C is a necessary cofactor in collagen biosynthesis; capillary fragility, which is a typical feature of scurvy, depends on the depletion of pericapillary collagen. As in our case, the earliest manifestations in patients with scurvy are at mucosal and cutaneous levels, with petechiae, ecchymoses, and gingival bleeding. Gastrointestinal manifestations are very rare in patients with scurvy. Submucosal hemorrhages involving the stomach, duodenum, and the colon, may cause gastrointestinal bleeding mimicking an inflammatory bowel disease.

Another typical hallmark of scurvy is anemia, which is multifactorial in its pathogenesis. In fact, it may be secondary to a combination of bleeding, decreased iron absorption, and other dietary deficiencies.

Finally, a further frequent manifestation in pediatric age is bone disease, which may be debilitating, as in our case. In fact it has to be underlined that our patient was hospitalized due to a clinical picture characterized by musculoskeletal pain and refusal to walk. These symptoms may be explained on the basis of a defect in osteoid matrix formation and cartilage reabsorption, leading to disordered bone structure and bone pain [5].

Radiologic studies can be useful for the diagnosis of scurvy, although the most specific signs are likely to be unfamiliar to many radiologists, owing to the rarity of this condition. The main radiographic findings are: white line of Fraenkel, which is an irregular, thickened white line that appears at the metaphysis and represents an increased calcification of the cartilage matrix; the Trummerfeld zone, which is a zone of rarefaction beneath the Fraenkel line, which denotes subperiosteal hemorrhage; Wimberger ring sign, which is a white line that surrounds ossification nucleus in the epiphysis; and the Pelkan sign, which is the presence of metaphyseal spurs that appear later due to the repair of microfractures [6].

Therefore, the diagnostic suspicion of scurvy is based on a combination of clinical signs and radiographic findings. The dosage of serum vitamin C levels is considered specific but laboratory tests are insensitive. It is known that serum concentrations do not always correspond with tissue storage of ascorbic acid. A reliable indicator of body storage is the measure of urinary excretion after intravenous ascorbic acid administration. Normally, 80% of absorbed vitamin C should be excreted within 3–5 hours. Lower levels of urinary excretion suggest vitamin deficiency [5]. Finally, the prompt resolution of symptoms after substitutive treatment represents the main evidence to confirm the diagnosis of scurvy [21].

The dose and duration of treatment should be individualized. It is demonstrated that ascorbic acid administration at an initial dose of 300 mg per day leads to complete resolution of symptoms within 4 weeks [1]. Then, vitamin C supplementation at a lower dose should be extended for another 2–3 months. Hemorrhagic manifestations, oral symptoms, and constitutional symptoms disappear within a very few days of starting the treatment, while the resolution of bone changes may take several weeks [22].

## Conclusions

Nowadays scurvy is a rare condition in industrialized countries and mainly appears in patients affected by underlying chronic disease. However, some case reports of apparent healthy children who developed scurvy have been reported in the literature over the last years. We point out the essential role of pediatricians in the eating habits of preschool children. The adoption of a detailed dietary anamnesis is fundamental to the early recognition of nutritional deficiency diseases and to avoid the performing of invasive procedures and/or their severe complications.

## Data Availability

Not applicable.

## Abbreviations

1,25-OH-vitamin D: 1,25-hydroxyvitamin D
25-OH-vitamin D: 25-hydroxyvitamin D
